# Book Review

**Published:** 1925

**Authors:** 


### Reviews of Books.

**Arteriosclerosis : A Summary View.** By the late Right Hon. Sir T. CLIFFORD ALLBUTT, P.C., K.C.B., M.A., M.D., etc. Pp. viii., 108. London : Macmillan & Co. Ltd. 1925. Price 5s.—This little book is worth a good deal more than many large monographs. In the first place, it expresses the considered views of the greatest English physician of his day on a subject to which he had given much thought. Secondly, it exemplifies wonderfully that combination of erudition with humility that made Sir Clifford Allbutt so great a teacher and so charming a man. Finally, it is his last work. As Sir Humphry Rolleston tells us in his prefatory note, it contains references to papers which appeared only a few weeks before the author's death. In its pages the work of men sixty years junior to its author receives a courteous and even respectful consideration, from a man in his ninetieth year, who speaks of Johnson, Gull and Sutton as his contemporaries. We doubt whether there is any finer example of the spirit of scholarship in medical literature, and as such we commend it to every student of medicine.

**The Chemical and Physiological Properties of the Internal Secretions.** By E. C. DODDS, Ph.D., B.Sc., M.B., B.S., and F. DICKENS, M.A., Ph.D. Pp. xii., 214. Oxford Medical Publications. 1925. Price 8s. 6d.—As the authors state in their preface, this book owes its publication to the fact that no single volume contains the chemical aspects of endocrinology, at least none written in this language. The collection of the many processes for isolation, synthesis and tests into one volume, it is hoped, would save researchers much time and translation. In order to catch the interest of the relatively numerous non-researchers, a summary of the physiological aspects was added. As regards their main object, the authors deserve congratulation ; this part is very full, and no omission of scientific importance has been noted. The absence of one hydrogen atom in the formula on page 127 appears to be a misprint. The physiological aspects, however, do not deserve the same praise : thus on the thyroid only one and a quarter pages are expended, and as a result much is omitted, even the relationship to tryptophane, a point which should appeal to chemists. On insulin the theory of Laufberger, which appears best able to explain its action, receives no mention ; another factor of importance and of interest to chemists, the role of hexose-phosphoric acid in the metabolism of glucose, is not noted. The unexplained action of ergotoxin (page 36) is what would be expected from the experimental work on this substance. The main action of tethelin, its growth stimulating action, would appear to be due to vitamin rather than definite hormone activity, if the work of Deutsch is correct. The mode of secretion of pituitrin, left as undetermined, scarcely deserves such a fate when the experimental work of Trendelenberg and Rémy Collin is put in the balance along with that of Cushing. The galactogogue action of pituitrin is regarded as being of clinical importance ; the work of Hall and Sutherland and others has shown that this action is purely temporary and of real value. Much recent work is not given attention ; thus, of the 120 references on the pituitry, only 31 date from 1918. Altogether the book is very ill-balanced ; the chemical features are relatively too full, and the physiological aspects are badly scamped, yet it is the latter which will pay for the publication. It is suggested that if a second edition is called for the lesser important chemical questions, many of which have only a problematical historical value, might be omitted to allow for the expansion of the really important scientific facts of the physiologists.

**Alcohol in Medical Practice.** By COURTENAY C. WEEKS, M.R.C.S., L.R.C.P. Pp. viii., 186. London : H. K. Lewis & Co. 1925. Price 3s. 6d. net.—Whilst the evolution of medical opinion in respect to the medicinal use of alcohol is the main theme of Dr. Weeks' book, there are also chapters devoted to the value of alcohol in pneumonia, in circulatory disease, in dyspepsia, in the diseases of children, in shock, and other surgical conditions. A most valuable series of appendices gives tables showing the amounts spent in alcohol by hospitals, infirmaries and sanatoria. The steady fall in the money expended on wines and spirits throughout hospitals in the United Kingdom is well illustrated, for instance, by the figures referring to our own General Hospital. In 1900 the total expenditure on 2,269 patients was £101, whilst in 1923, with 4,450 patients, and in spite of the greatly increased price of liquors, the expenditure was £78. The very wide variation in the amount of alcohol given in hospitals, which should be fairly comparable, shows, however, that there is much promiscuous prescribing, and that alcohol is not scientifically administered in the way a potent drug should be. The old tradition which regarded alcohol as a stimulant is slowly dying out, but further investigation is needed to ascertain its true value as a narcotic and as a food. Dr. Weeks does not conceal his own views as to the use and abuse of alcohol, but he has written in a broad

19

and scientific spirit, and his book will be read with great profit by all students and practitioners of medicine. It is full of valuable information and reference to recent scientific work on alcohol, and will do much to influence medical teaching and medical practice.

**Vital Capacity of the Lungs.** By J. A. MYERS, M.S., Ph.D., M.D. Pp. 119. Baltimore: The Williams & Wilkins Co. 1925. Price 16s. 6d. net.—Dr. Myers has done well to put together this short account of the measurement of the vital capacity of the lungs and its application to clinical medicine. The book can be read in the course of an hour or so. There is a good list of references. The price seems rather excessive.

**Abdominal and Pelvic Surgery for Practitioners.** By RUTHERFORD MORISON, Hon. M.A. and D.C.L., Hon. LL.D., M.B., F.R.C.S. (Ed. and Eng.). Pp. x., 212. Humphrey Milford, Oxford University Press. 1925. Price 8s. 6d. net.—What Rutherford Morison writes is always well worth reading, and this small and unpretentious book is no exception to this statement. Its title perhaps is a little misleading, because one usually expects a book dealing with Surgery to be concerned mainly with treatment and operations. But this is intended as a guide to diagnosis, so that dangerous or fatal delay in the recognition of disease may not occur. This early diagnosis the author holds should be the function of the general practitioner, and not be left solely in the hands of specialists, who are often not consulted until the patient has become aware of some serious disease. The book is divided into three sections dealing with emergencies, subacute abdominal disease and chronic diseases. Methods of examination and the interpretation of symptoms form the main themes, and every type of disease is aptly illustrated by examples taken from the author's own practice. At the end of the book are short sections dealing with the scope and general results of abdominal operations and the after-care of the patients. These might well have been longer.

**The Crippled Hand and Arm.** By CARL BECK, M.D. Pp. xi., 243. London: J. B. Lippincott Co. 1925. Price 30s. net. —The book is nicely printed and illustrated, and it deals with a subject of great importance. A hand or arm crippled by injury or disease is a terrible disaster, and the restoration of its function is of the utmost importance both to the individual and to the community at large. The author's methods of effecting this are ingenious, and will repay study. Before the reader can grasp them, however, study will undoubtedly be required, for the book is written in English so faulty and a style so obscure that the reviewer has often been tempted to cast it aside in despair. In his opinion it can never obtain any circulation in this country till it is adequately translated and more lucidly written.

**Synopsis of Medicine.** By H. Letheby Tidy, M.A., M.D., B.Ch. (Oxon.), F.R.C.P. (Lond.). Fourth edition. Pp. xv., 1,000. Bristol: John Wright & Sons Ltd. 1925. Price 21s. net.—The fourth edition of this valuable synopsis contains among its changes an extensively re-written chapter on Diabetes Mellitus, dealing fully with the treatment of this disease by Insulin, and practically new articles on Botulism, Visceroptosis, Hæmatoporphyrinuria, Infarction of the Lung, Primary Purpura, and Acholuric Jaundice. There are important alterations too in the chapters on Gall-stones and Bronchial Asthma. Blood Diseases of Childhood form the subject of a new chapter. There are other alterations of a minor character, but they all serve to maintain the high position which this *Synopsis of Medicine* already holds as an up-to-date guide and epitome. We have few criticisms to offer, but in the treatment of Diabetes Mellitus we are inclined to think that almost too much stress is laid on the "fasting" method. Barting's advice that the "starvation" period should be reduced to a minimum might have been mentioned as a view worthy of consideration, even though it conflicts with the views of Allen and Graham. Taken as a whole, Tidy's *Synopsis* fully deserves its popularity among practitioners and students.

**A Synopsis of Surgery.** By Ernest W. Hey Groves, M.S., M.D., B.Sc. (Lond.), F.R.C.S. (Eng.). Seventh edition. Pp. viii., 671. Bristol: J. Wright & Son Ltd.—This thoroughly up-to-date edition will still further enhance the reputation of this unrivalled and complete surgical hand-book. The general arrangement is unaltered, but there is added a wealth of the latest information in the present publication. Certainly, in the space allotted to them, it would be hard to better the chapters on biliary surgery and the acute abdomen, whilst the instructive scheme for testing urinary function marks a real advance as a scientific gauge of bodily function, and is superior to the loose handling this modern subject receives in the larger text-books. The text is illuminated by numerous drawings and diagrams which are a new and notable addition. Most comprehensive is the series of illustrations representing modern methods of dealing with the fractured femur. A chapter of clear diagrams of well-chosen surface markings make an attractive finish to a fine work. Certainly *A Synopsis of Surgery* by Hey Groves starts the student on the right road for the summit of surgery.

**Artificial Sunlight and its Therapeutic Uses.** By FRANCIS HOWARD HUMPHRIS, M.D. (Brux.), F.R.C.P. (Edin.). Second edition. 1925. Pp. xvi., 203. Oxford Medical Publications. Price 8s. 6d.—The second edition of this excellent treatise on the nature of "Light Treatment," and the type of diseases which may be benefited, follows hard after the first. This alone shows that in the widening interest taken in the various forms of lamps available for the production of therapeutic light the profession has welcomed a book which explains in simple language, without dogmatism or exaggeration, the types of lamp at present in use, and the kind of results which may be expected from them.

**A Preliminary Report on the Treatment of Interstitial Keratitis.** By R. LINDSAY REA, M.D., F.R.C.S. Pp. 32. London: H. K. Lewis & Co. Ltd. 1925. Price 2s. 6d.—This little book gives a brief account of ninety-one cases of interstitial keratitis treated by the author, with details of the methods employed. The cases were all syphilitic in origin. It is interesting to note, that of eight cases in which the Wassermann reaction was negative five gave a luetic reaction when tested by Lange's colloid gold test. The author warns observers against labelling such cases as tuberculous without having this test done. He makes out a strong case for treating these cases by injection with one or other of the arsenical preparations, followed by hyd. c. creta. He prefers novarsenobillion or, in the case of very young children, sulfarsenol, given intramuscularly if intravenous injections are not possible. He does not claim that the results are dramatic, but contends that, judged by the vision resulting after treatment, this method constitutes a real advance on the older methods of treatment. He mentions the significant fact that not one of the 64 new cases treated by him from the outset developed deafness. It is true that some writers say that treatment either by mercury or by the newer methods is useless in these cases. There is, however, an increasing amount of evidence in the literature that treatment by the newer methods is of value. We have seen a few cases markedly benefited by treatment with the arsenical preparations; too few to enable us to form a proper opinion as to their value generally, but enough to convince us as to their value in some cases at least. We shall, therefore, look forward to seeing a second edition of this book, based on a further series of cases. The book, which is well printed, contains an interesting frontispiece showing the contrast between a syphilitic child and her mother; also three useful plates, two of which are in colour. It is a book well worth reading.

**Annals of the Pickett-Thomson Research Laboratory.** Vol. I., Part 2. June, 1925. London : Baillière, Tindall & Cox.—The second part of these Annals contains the results of inquiries upon the preparation of high-class nutrient media for germs which are difficult to grow, upon bacterium pneumosintes, the bacteriology of the respiratory tract in cases of influenza, measles, etc., with the discovery of six new species of organisms and the classification and identification of germs by micro-photography. The whole of the work has been carried out by Robert and David Thomson. It evidences a mass of untiring energy and ingenuity. The authors have paid special attention to the value of media prepared from testicular tissues, and have investigated the characters and usefulness of various blood preparations. Among the latter they employed sterile filtered blood which had been allowed to putrefy, viz. had been changed in character by the action of bacteria. The result was so startling that it is suggested that the products of sepsis entering the blood from decayed teeth or from intestinal toxæmia go to favour the suitability of the living body as a host for such dangerous organisms, and render the individual more susceptible to attacks by them. There is a good account of the bacterium pneumosintes and allied respiratory organisms. It may be of interest to the general reader to learn that it sometimes requires three months' daily careful nursing of cultures before the specific organism can be isolated and grown in pure culture by itself. In Switzerland, especially during the winter sport season, a virulent form of epidemic fever often attacks the visitors. The local doctors consider it to be influenza. It is particularly rife in the Engadine, and has been termed Engadine fever. The germs present in the sputum from such cases belong to the pneumosintes group. There are, however, quite a number of bacteria demonstrable in respiratory diseases by use of these new methods of culture. Their actual pathogenetic properties are not yet determined. The volume closes with a number of micro-photographs of bacterial cultures at various stages of growth, and illustrations of mechanical devices for grinding tissues and bacteria.

**A Text-Book for Nurses.** By E. W. Hey Groves, M.D., B.Sc., M.S., F.R.C.S., and the late J. M. Fortescue Brickdale, M.A., M.D. (Oxon.), M.R.C.P. (Lond.). The Medical Section revised by J. A. Nixon, C.M.G., M.D. (Cantab.), F.R.C.P. (Lond.). Pp. xxx., 645. Third edition. 1925. Oxford Medical Publications. Price 20s.—The keynote of this, the third edition of this popular book, is contained in a sentence in the original Preface, which runs, " The chief aim . . . is to enable the nurse to understand the principles underlying the Medical and

Surgical treatments which it is her duty to assist in carrying out." In these days of increased theoretical teaching there is, without doubt, great danger that the nurse may learn her work parrot fashion, and this book is an excellent antidote to that danger. The descriptions are clear, the reasoning simply set out, and the letterpress and illustrations are both excellent. It is unusual to find Anatomy, Physiology, the description and treatment of disease or injury, and a comprehensive chapter on Hygiene, all contained in one book ; it is certainly, from the point of view of teachers of nurses and of the nurses themselves, one of the most convenient publications of recent times. By what seems to be a curious error, the heading of each left-hand page is "Text-book of Nursing," a description which is inaccurate.

**Practical Chiropody.** By E. G. V. Runting, F.I.S.Ch. Pp. ix., 164. London : The Scientific Press Ltd. 1925. Price 5s. net.—Painful affections of the feet are among the commonest of complaints in civilised races. They cause great disability, and it is small wonder that medical advice is often sought with a view to obtaining relief. Practical Chiropody is a small book, on the treatment of corns, warts, ingrowing toe-nail, etc. It is simply written, and is evidently primarily intended for the benefit of chiropodists, but the general practitioner may pick up many useful hints from its perusal. If he knows how to cure a painful corn he will be certain of many grateful patients.

**C.M.B. Examination Questions and Model Answers. A Handbook for Nurses entering for the Central Midwives' Board Examination, with Abridged Rules.** Second edition. Pp. 150. London : The Scientific Press Ltd. 1925. Price 1s. 6d. net.—A very excellent little text-book, invaluable to those nurses going up for their C.M.B. examination.

**Gynæcology for Nurses and Gynæcological Nursing.** By Comyns Berkeley, M.A., M.D., M.C. (Cantab.), F.R.C.P. (Lond.), M.R.C.S. (Eng.). Pp. xi., 364. London : The Scientific Press Ltd. 1925. Price 7s. 6d. net.—A useful book of reference for those nurses who specialise in gynæcological nursing, but much too full for the needs of nurses taking the General Nursing Council examination. A large amount of midwifery teaching is included, and in consequence the book would be useful for the C.M.B. nurse both before and more especially after she has obtained her certificate.

**Canned Foods in relation to Health.** (Milroy Lectures, 1923.) By William G. Savage, B.Sc., M.D. (Lond.), D.P.H. Pp. vi., 146. Cambridge : University Press. 1923. Price 8s. 6d. net,

—Dr. Savage has condensed a very large scattered literature and the results of his own investigations into a reasonably small volume. He deals with the chemical and bacteriological contaminations and the processes of canning chiefly, but changes occurring during maturation and notes on vitamin persistence are included. He also outlines methods for laboratory examination, and suggests means of control calculated to ensure greater purity. There is a bibliography. A greater knowledge on this subject is urgently needed. The poorer sections of the community subsist very largely on these preserved, ready-cooked foods. As they are relatively cheap, and save time and expense in preparation, we may expect that they will continue to be used. That diet is a great factor in the production of good or bad health is such a truism that it tends to be disregarded; by a slight variation in the diet bees can manufacture neuters or queens at will. It may well be that diet in man makes the difference between an A1 and a C3 individual. A point arises which does not appear to be treated: the question of the biological values of these prepared foods, if, as it would appear, this is not merely due to the vitamin content. Can we be assured that 100 calories of a preserved food is equal to 100 calories of the same fresh food? Also we are told that the danger of tin poisoning is trivial; proof that the continuous ingestion of minute amounts of tin is without any effect would be welcome. Dr. Savage is to be congratulated on his work, and it is hoped that research in this subject will continue.

**A Text-Book of Pathology, General and Special.** By J. Martin Beattie, M.A. (N.Z.), M.D. (Edin.), M.R.C.S., L.R.C.P. (Lond.), and W. E. Carnegie Dickson, M.D., B.Sc., F.R.C.P. (Edin.). London: William Heinemann.—This well-known text-book is now published in one heavy volume; the text remains the same except for minor additions. One method of selecting a text-book of pathology from the standard works at present at the student's disposal is to weigh each and select the lightest. We believe that many students preferred this treatise in two parts as in the second edition, and that the issue of a single weighty volume may drive them to purchasing a lighter and equally reliable book. From the nature of its inquiries, pathology will always remain the most difficult subject in the curriculum, and the choice of a text-book, provided its teaching is reliable, as that of Beattie and Dickson assuredly is, is a matter of taste. We frequently hear students complaining that, having bought a pathological text-book, they cannot read it at all or they do not read it easily. We would counsel them to read the same subject in this and in, say, three other standard text-books before adopting any book as their guide, philosopher and friend. The interesting speculations and excellent diagrams of MacCallum will appeal to some, the conciseness of Muir to others, and the comforting, non-committal nature of the discussion and the careful recording of fact of Beattie and Dickson to others. Some of the plates in this edition are showing signs of wear.

**Text-Book of Surgical Pathology.** By C. Jennings Marshall, M.D., M.S. (Lond.), F.R.C.S. (Eng.), and Alfred Piney, M.D., Ch.B. (Birm.), M.R.C.P., M.R.C.S. Pp. vii., 469. London: Edward Arnold & Co. Price 21s. net.—The authors of this book of about 500 pages have set themselves the task of correlating "embryology, anatomy, pathology and clinical observation" in order to help the student of surgery. They have succeeded in compressing into these 500 pages a large mass of information which otherwise could only be obtained by the use of several text-books. The descriptions follow a strictly clinical sequence. Much space is devoted to morbid anatomy and embryology, and the student is kept closely in touch with clinical surgery. The pathological author has not allowed himself to speculate at any length on "the causes of things": microscopic descriptions are short and, to the inquiring mind, disappointing. The style is necessarily rather telegraphic, and far from restful. Many of the illustrations, especially those dealing with diseases of bone and the line diagrams illustrating development, are very good; others, however, are disappointing, and a few are misleading. It is obviously difficult to provide the modern over-worked medical student with a volume which will be, at the same time, interesting to read and useful for examinations, well illustrated and cheap; and there is no doubt that for students preparing for the higher surgical examinations, and for those whose interests and ambitions are mainly surgical, this book will prove of considerable use, but there is more of interest in pathology as applied to surgery than this book indicates.

**The Pathology of Tumours.** By E. H. Kettle, M.D., B.S. (Lond.). Second edition. Pp. viii., 285. London: H. K. Lewis & Co. Ltd. 1925. Price 12s. 6d. net.—This book can be cordially recommended to the student who wishes to read in the compass of 275 well-written pages the best complete account of the pathology of tumours written by an English pathologist. The illustrations are wonderfully clear, the text concise and easy to read, while this edition is enriched by new illustrations of naked-eye appearances. British pathology should be proud of the book. |

